# Presence of *Spirometra mansoni*, Causative Agent of Sparganosis, in South America

**DOI:** 10.3201/eid2811.220529

**Published:** 2022-11

**Authors:** Jan Brabec, Manuel Uribe, Jenny J. Chaparro-Gutiérrez, Carlos Hermosilla

**Affiliations:** Centre of the Czech Academy of Sciences, České Budějovice, Czech Republic (J. Brabec);; CIBAV Research Group, Universidad de Antioquia, Medellín, Colombia (M. Uribe, J.J. Chaparro-Gutiérrez);; Justus Liebig University Giessen, Giessen, Germany (M. Uribe, C. Hermosilla)

**Keywords:** sparganosis, Spirometra mansoni, South America, parasites, zoonoses, Colombia

## Abstract

We report molecular identification of an adult *Spirometra mansoni* tapeworm retrieved from a crab-eating fox (*Cerdocyon thous*) in Colombia, confirming presence of this parasite in South America. This tapeworm is the causative agent of human sparganosis, commonly reported from Southeast Asia, and represents the second congeneric species with known zoonotic potential in the Americas.

Sparganosis is a neglected human zoonosis caused by migrating larval stages of the broad tapeworm genus *Spirometra* (Diphyllobothriidea), whose natural definitive hosts include wild and domestic canids and felids. The life cycle of this tapeworm involves 2 intermediate hosts: a freshwater copepod crustacean as the first and various vertebrates, mostly amphibians, as the second. Human infections are commonly reported from Southeast Asia and propagate most often in the form of subcutaneous sparganosis; however, the larvae can enter other organs or parts of central nervous system and cause damage.

Taxonomy of *Spirometra* remains highly complicated. Numerous species of *Spirometra* have been described, often poorly (*1*), and representatives of just 6 species-level lineages have been characterized molecularly so far, a key prerequisite to achieve a convincing tapeworm identification when only strobila fragments or larval stages are available. Limitations of morphologic characters of *Spirometra* are numerous and include characters’ great intraspecific and even intra-individual variability (overview of problematic traits in *2*). Molecular sequence data thus represent the only unequivocal method of species identification.

Previous phylogenetic analysis of *Spirometra* has shown that the geographic distribution of the 6 lineages respects continental borders (*2*). North and South America were shown to share 2 lineages found exclusively on those continents (*3*), provisionally termed *Spirometra decipiens* complex 1 and 2 because of the lack of essential morphologic data precluding conclusive species determination (*2*). *S. decipiens* complex 1 was shown to house, among parasites of canids and felids, causative agents of cutaneous and proliferative sparganosis. Representatives of *S*. *decipiens* complex 2, on the other hand, have not yet been shown to cause the zoonosis. The frequently reported human cases of sparganosis from Southeast Asia, as well as numerous specimens from wildlife from the region, corresponded to *S*. *mansoni* (*2*).

We report molecular identification of a tapeworm specimen retrieved from a dead crab-eating fox (*Cerdocyon thous*) from the vicinity of Ciudad Bolívar, Antioquia, Colombia. We characterized the specimen through Sanger-sequencing of 3 genetic loci (Appendix), including the complete mitochondrial cytochrome c oxidase subunit I gene (*cox1*) as the most densely sampled and phylogenetically informative gene of broad tapeworms. Phylogenetic analysis under maximum-likelihood criterion resolved the position of the tapeworm nested deep within the clade of *S*. *mansoni* (Figure), proving the presence of this causative agent of human sparganosis on the American continents.

**Figure:**
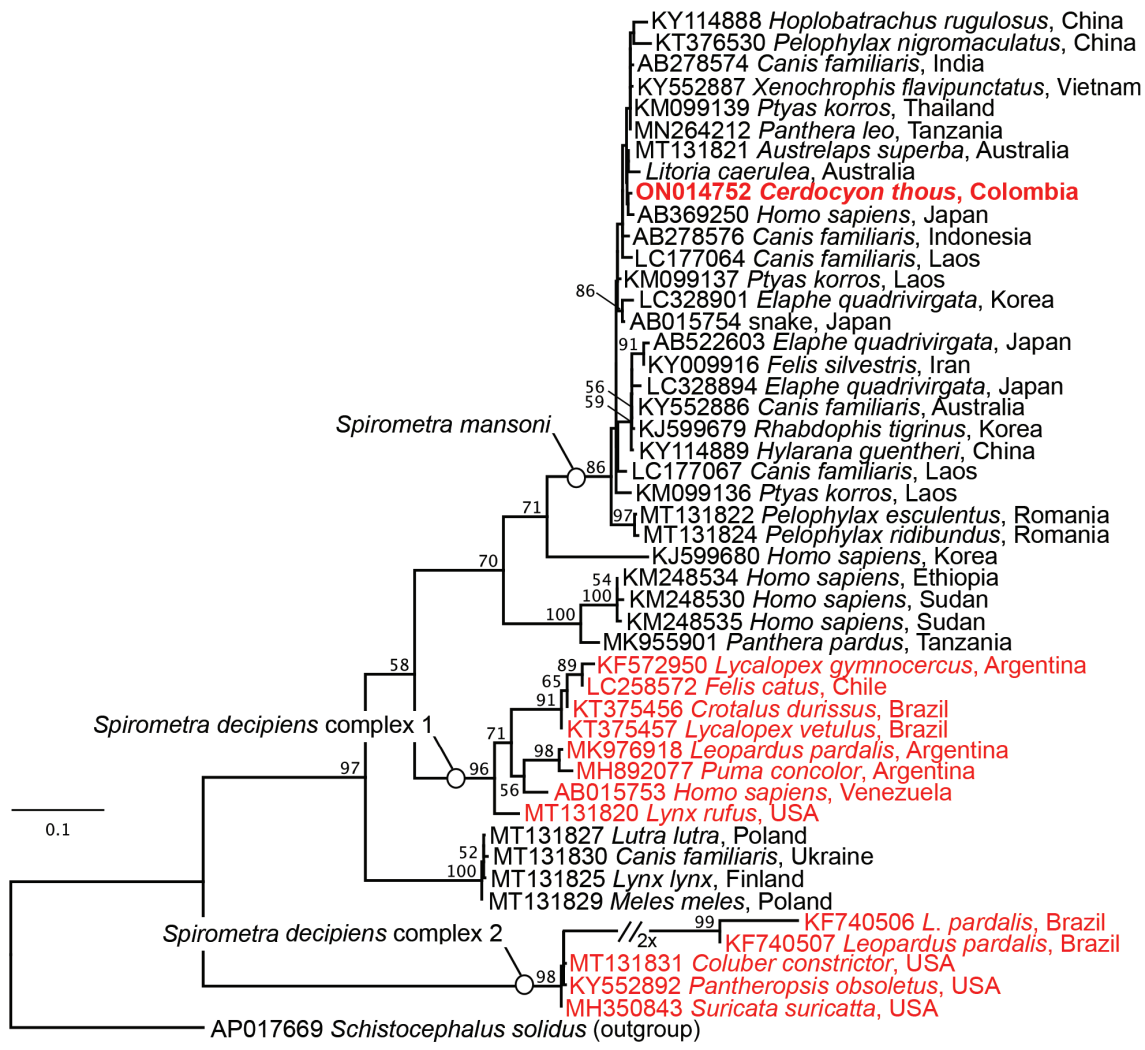
Maximum-likelihood estimate of the phylogenetic position of a *Spirometra* *mansoni* tapeworm collected from a crab-eating fox (*Cerdocyon thous*) in Colombia. Red indicates specimens from South America; bold indicates newly characterized *S. mansoni* from this report. Names of the 3 species-level lineages of *Spirometra* in South America are indicated; GenBank numbers are provided. Nodal support values show standard bootstrap supports >50. Scale bar indicates number of substitutions per site.

*S. mansoni* represents by far the most frequently reported causative agent of sparganosis, previously misidentified as *S*. *erinaceieuropaei* (*2*). This species is responsible for virtually all human cases in Asia but has been also shown to infect wildlife in Africa, Australia, and Eastern Europe (*2*). Our finding of *S*. *mansoni* in Colombia in a crab-eating fox, a definitive host endemic and widely distributed across South America, from Panama to the Entre Ríos province of Argentina (*4*), expands the known distribution of *S*. *mansoni* into broader range than previously thought. This finding contrasts with the distribution of the remaining 5 lineages of *Spirometra*, which seem limited to continental regions (*2*). *S. mansoni* has been sporadically reported from the Americas in the past; however, morphology-altering fixation techniques and lack of critical molecular evidence did not support species identification. Reported hosts mostly included domestic cats (Appendix) and a single report from a crab-eating fox in Brazil (*5*).

The crab-eating fox inhabits savannah and woodland areas of various Neotropical habitats from coastal plains to montane forests and is considered omnivorous, opportunistically feeding on fruits, insects, and small vertebrates including amphibians and reptiles, with seasonal shifts to its diet (*6*,*7*). A broad range of Neotropical amphibians and reptiles has been found to serve as intermediate hosts of *Spirometra*; however, the record remains skewed toward herpetofauna of the more intensively surveyed coastal regions (*8*), and species identification of the parasite larvae has been, thanks to the lack of accompanying molecular data, either absent or ungrounded. As a result, the real range and the relevance of different intermediate hosts for the transmission of the sympatric South America species of *Spirometra* remain unknown. The situation in North America is even more obscure because of the virtually missing intermediate host record (*1*,*9*). Given the wide spectrum of suitable intermediate hosts of *S*. *mansoni*, which includes omnivores such as wild boar in Europe (*10*), the natural pools and the importance of different host species in the etiology of the zoonosis remain dubious. The concurrent presence of the second congeneric species with zoonotic potential urges deeper investigations into the parasite’s life cycles and the epizootiology of a disease that could affect public health in the Americas.

Appendix Additional historical reports of *Spirometra mansoni* from domestic cats mentioned in the main text include Puerto Rico, Chile and Costa Rica
