# Supplementary material for: Presence of Spirometra mansoni, Causative Agent of Sparganosis, in South America
Source: Emerg Infect Dis. 2022 Nov;28(11):2347–50. doi: 10.3201/eid2811.220529 (PMC9622250; doi:10.3201/eid2811.220529)
Supplement: Appendix — Additional historical reports of Spirometra mansoni from domestic cats mentioned in the main text include Puerto Rico, Chile and Costa Rica [file 22-0529-Techapp-s1.pdf]

Article DOI: http://doi.org/10.3201/eid2811.220529

Presence of *Spirometra mansoni*, Causative Agent of Sparganosis, in South America

Appendix

Additional historical reports of *Spirometra mansoni* from domestic cats mentioned in the main text include Puerto Rico, Chile and Costa Rica (*1*,*5*,*7*).

Methods

Under the scope of a survey of parasites of Colombian canids, we carried out a necropsy of an adult male crab-eating fox (*Cerdocyon thous*) found at 1,084 meters above sea level in the Andean municipality of Ciudad Bolívar, Antioquia, Colombia (5°52′02″N, 75°57′15.42″W). During detailed examination of the carcass and cavitary organs we recovered an entire cestode specimen and a strobila fragment from the duodenum. The two strobila fragments were rinsed in saline and preserved in 90% ethanol, unwittingly of the fixative's detrimental effects on conserving the soft tissue quality and associated morphological characteristics necessary for morphological identification and morphometrics.

Total genomic DNA was extracted from snippets of the two ethanol-preserved strobila pieces using the Monarch Genomic DNA Purification kit (New England Biolabs, Inc.) following the manufacturer's protocol. The complete sequence of the *cox1* gene (1,566 bp) was PCR amplified with primers Cox1Forward and Cox1Reverse of Wicht et al. (*7*) using the Phusion High-Fidelity DNA Polymerase (New England Biolabs, Inc.) and the following cycling conditions: 35 cycles of 10 s at 98 °C, 15 s at 53 °C, 45 s at 72 °C. Nearly complete sequence of the nuclear small subunit ribosomal RNA gene (ssrDNA) was amplified over 30 cycles of 10 s at 98 °C, 15 s at 58 °C, 60 s at 72 °C using the primers WormA and WormB. Partial nuclear large subunit ribosomal RNA gene (lsrDNA) was amplified over 30 cycles of 10 s at 98 °C, 15 s at 63 °C, 45 s at 72 °C using the primers LSU5 and 1500R.

PCR products were gel-checked and enzymatically purified with Exonuclease I and FastAP alkaline phosphatase (Thermo Fisher Scientific). Sanger sequencing was carried out by SeqMe (Czech Republic) using the PCR as well as the following internal primers:

Cox1Forward (TATCAAATTAAGTTAAGTAGACTA) **cox1**

Cox1Reverse (CCAAATAGCATGATGCAAAAG) **cox1**

JB3 (TTTTTTGGGCATCCTGAGGTTTAT) **cox1**

JB4.5 (TAAAGAAAGAACATAATGAAAATG) **cox1**

WormA (GCGAATGGCTCATTAAATCAG) ssrDNA

WormB (CTTGTTACGACTTTTACTTCC) ssrDNA

600R (AACCGCGGCKGCTGGCACC) ssrDNA

1270F (ACTTAAAGGAATTGACGG) ssrDNA

1270R (CCGTCAATTCCTTTAAGT) ssrDNA

LSU5 (TAGGTCGACCCGCTGAAYTTAAGCA) lsrDNA

1500R (GCTATCCTGAGGGAAACTTCG) lsrDNA

ECD2 (CTTGGTCCGTGTTTCAAGACGGG) lsrDNA

900F (CCGTCTTGAAACACGGACCAAG) lsrDNA

400R (GGCAGCTTGACTACACCCG) lsrDNA

Contiguous gene sequences were assembled and inspected for errors in Geneious Prime 202.0.5 (http://www.geneious.com). The complete *cox1* sequences of the two strobila fragments were compared and found identical, suggesting their common origin from a single tapeworm individual. Newly characterized gene sequences were deposited in GenBank under accession nos. ON014752, ON016172 and ON032355.

The complete c*ox1* sequence was aligned with 47 representatives of the global genetic diversity of *Spirometra*, using the G-INS-i algorithm of the program MAFFT (*2*) implemented in Geneious. Uncharacterized parts of the partial sequences downloaded from GenBank were encoded as missing data. The maximum likelihood tree was estimated under the following best-scoring model and partitioning scheme TIM3+F+I+G4: part1, TN+F+I: part2, TIM2+F+R3:

part3 selected by ModelFinder (*3*) according to the corrected Akaike information criterion in the program IQ-TREE (*4*). Nodal support values were estimated through running 100 standard nonparametric bootstrap resamples in IQ-TREE. Pairwise sequence similarity of the newly characterized *cox1* to the remaining representatives of *S. mansoni* ranged between 99.49 and 96.55 %.

References

1. Cram EB. *Diphyllobothrium mansoni* in Porto Rican cat. J Parasitol. 1927;1:70.

2. Katoh K, Standley DM. MAFFT multiple sequence alignment software version 7: improvements in performance and usability. Mol Biol Evol. 2013;30:772–80. PubMed https://doi.org/10.1093/molbev/mst010

3. Kalyaanamoorthy S, Minh BQ, Wong TKF, von Haeseler A, Jermiin LS. ModelFinder: fast model selection for accurate phylogenetic estimates. Nat Methods. 2017;14:587–9. PubMed https://doi.org/10.1038/nmeth.4285

4. Nguyen L-T, Schmidt HA, von Haeseler A, Minh BQ. IQ-TREE: a fast and effective stochastic algorithm for estimating maximum-likelihood phylogenies. Mol Biol Evol. 2015;32:268–74. PubMed https://doi.org/10.1093/molbev/msu300

5. Torres P, Figueroa L. Infección por *Spirometra mansoni* (Cestoda, Pseudophyllidea) en el sur de Chile. Bol Chil Parasitol. 1982;37:72–3. PubMed

6. Valerio I, Rodríguez B, Chinchilla M. Primer hallazgo de *Spirometra mansoni* en *Felis domesticus* de Costa Rica. Parasitol Latinoam. 2004;59:162–6. https://doi.org/10.4067/S0717-77122004000300013

7. Wicht B, Yanagida T, Scholz T, Ito A, Jiménez JA, Brabec J. Multiplex PCR for differential identification of broad tapeworms (Cestoda: *Diphyllobothrium*) infecting humans. J Clin Microbiol. 2010;48:3111–6. PubMed https://doi.org/10.1128/JCM.00445-10
